# Despite phylogenetic effects, C_3_–C_4_ lineages bridge the ecological gap to C_4_ photosynthesis

**DOI:** 10.1093/jxb/erw451

**Published:** 2016-12-26

**Authors:** Marjorie R Lundgren, Pascal-Antoine Christin

**Affiliations:** Department of Animal and Plant Sciences, University of Sheffield, Western Bank, Sheffield, UK

**Keywords:** Biomes, C_3_–C_4_ intermediate, C_4_ photosynthesis, ecology, evolution, phylogeny

## Abstract

C_4_ photosynthesis is a physiological innovation involving several anatomical and biochemical components that emerged recurrently in flowering plants. This complex trait evolved via a series of physiological intermediates, broadly termed ‘C_3_–C_4_’, which have been widely studied to understand C_4_ origins. While this research program has focused on biochemistry, physiology, and anatomy, the ecology of these intermediates remains largely unexplored. Here, we use global occurrence data and local habitat descriptions to characterize the niches of multiple C_3_–C_4_ lineages, as well as their close C_3_ and C_4_ relatives. While C_3_–C_4_ taxa tend to occur in warm climates, their abiotic niches are spread along other dimensions, making it impossible to define a universal C_3_–C_4_ niche. Phylogeny-based comparisons suggest that, despite shifts associated with photosynthetic types, the precipitation component of the C_3_–C_4_ niche is particularly lineage specific, being highly correlated with that of closely related C_3_ and C_4_ taxa. Our large-scale analyses suggest that C_3_–C_4_ lineages converged toward warm habitats, which may have facilitated the transition to C_4_ photosynthesis, effectively bridging the ecological gap between C_3_ and C_4_ plants. The intermediates retained some precipitation aspects of their C_3_ ancestors’ habitat, and likely transmitted them to their C_4_ descendants, contributing to the diversity among C_4_ lineages seen today.

## Teaser

The C_3_–C_4_ state moves lineages into C_4_-like environments, bridging the ecological gap between C_3_ and C_4_ species and facilitating C_4_ evolution.

## Introduction

The C_4_ photosynthetic pathway relies on a coordinated system of anatomical and biochemical traits that function to concentrate CO_2_ around Rubisco, which in most C_4_ plants is localized to the bundle sheath cells (Hatch, 1987). The enhanced CO_2_ concentration substantially suppresses O_2_ fixation and subsequent photorespiration, compared with the ancestral C_3_ photosynthetic pathway, making C_4_ photosynthesis advantageous in conditions that increase photorespiration (Chollet and Ogren, 1975; Hatch and Osmond, 1976). C_4_ photosynthesis is consequently prevalent in the open biomes of warm regions where it boosts growth (Sage *et al.*, 1999; Osborne and Freckleton, 2009; Atkinson *et al.*, 2016), to ultimately shape entire ecosystems, such as the emblematic savannas (Sage and Stata, 2015).

It has been widely reported that some plants possess only a subset of the anatomical and/or biochemical components of the C_4_ pump. These plants tend to be physiologically somewhere in between typical C_3_ and C_4_ plants and, as such, are termed C_3_–C_4_ intermediates (Kennedy and Laetsch, 1974; Monson and Moore, 1989; Sage, 2004; Schlüter and Weber, 2016). These physiologically intermediate plants use a photorespiratory CO_2_ pump, or glycine shuttle, to rescue CO_2_ released from mesophyll photorespiratory activity and transport it into the bundle sheath for re-use in the Calvin cycle located there (Hylton *et al.*, 1988). Thus, the C_3_–C_4_ system establishes a CO_2_ recycling mechanism based on the spatial segregation of metabolic reactions, the migration of the Calvin cycle to the bundle sheath, and the dual-compartment coordination that are characteristic of the C_4_ pathway. These modifications improve the physiological performance of C_3_–C_4_ plants over the C_3_ system in conditions that promote photorespiration, as they lessen the total carbon lost via photorespiration to improve net carbon assimilation (Vogan and Sage, 2011; Way *et al.*, 2014). In addition to the glycine shuttle, a number of C_3_–C_4_ plants engage a weak C_4_ cycle (Ku *et al.*, 1983), which further reduces photorespiration and is predicted to increase biomass accumulation (Mallmann *et al.*, 2014). Thus, this variation in C_4_-associated traits forms a continuum between the C_3_ condition and a diversity of C_4_ phenotypes (Bauwe, 1984; McKown and Dengler, 2007; Lundgren *et al.*, 2014; Bräutigam and Gowik, 2016).

Because C_3_–C_4_ plants share many anatomical, biochemical, and physiological traits with C_4_ plants, they are often assumed to represent an evolutionary step facilitating C_4_ evolution (Hylton *et al.*, 1988; Sage, 2004; Sage *et al.*, 2012; Bräutigam and Gowik, 2016), a hypothesis confirmed by the close relationships between C_3_–C_4_ and C_4_ taxa in some groups (McKown *et al.*, 2005; Christin *et al.*, 2011*b*; Khoshravesh *et al.*, 2012; Sage *et al.*, 2012; Fisher *et al.*, 2015). They are consequently widely studied and incorporated into models of C_4_ evolution, which show that C_3_–C_4_ phenotypes can bridge the gap between C_3_ and C_4_ states by providing a series of stages that are advantageous over the preceding ones (Heckmann *et al.*, 2013; Williams *et al.*, 2013; Mallmann *et al.*, 2014; Bräutigam and Gowik, 2016). This research program has been extremely successful in tracking the changes in leaf anatomy, organelles, metabolism, genes, and enzymes that likely took place during C_4_ evolution, particularly in the eudicot genus *Flaveria* (e.g. Bauwe and Chollet, 1986; Svensson *et al.*, 2003; McKown and Dengler, 2007, 2009; Sage *et al.*, 2013). However, previous research failed to address the ecological consequences of these intermediate stages. Indeed, while models that predict the carbon gains of the intermediate stages exist (Heckmann *et al.*, 2013; Mallmann *et al.*, 2014), studies of natural distributions of extant C_3_–C_4_ taxa are nearly non-existent (but see Sudderth *et al.*, 2009).

The differing geographical and environmental distributions of C_3_ and C_4_ species have been widely studied (Teeri and Stowe, 1976; Rundel, 1980; Williams *et al.*, 1995; Ehleringer *et al.*, 1997, Epstein *et al.*, 1997; Edwards and Still, 2008), with later incorporation of phylogenetic data providing estimates of the ecological shifts that happened before, during, or after photosynthetic transitions (Osborne and Freckleton, 2009; Edwards and Smith, 2010; Edwards and Ogburn, 2012; Kadereit *et al.*, 2012; Lundgren *et al.*, 2015). However, these efforts focused on comparisons between C_3_ and C_4_ plants, which are much more frequent and abundant than C_3_–C_4_ taxa. Previous discussions of C_3_–C_4_ ecology characterized their distributions in hot, sandy, and disturbed habitats with little competition (Powell 1978; Hedge and Patil, 1980; Prendergast and Hattersley, 1985; Vogan *et al.*, 2007; Feodorova *et al.*, 2010; Christin *et al.*, 2011*b*; Sage *et al.*, 2011, 2012). However, other groups with C_3_–C_4_ intermediates thrive in apparently very different habitats, with C_3_–C_4_*Flaveria* inhabiting a broad range of environments from open fields and scrublands (*F. angustifolia*) to pine forests (*F. anomala*), wetlands (*F. floridana*), and warm mineral springs (*F. sonorensis*; Powell 1978), yet field data failed to identify differences in the distributions of different photosynthetic types in *Flaveria* (Sudderth *et al.*, 2009). The monocot C_3_–C_4_ intermediates of *Eleocharis* and *Steinchisma* thrive in wetland habitats (USDA/NRCS, 2016), C_3_–C_4_*Alloteropsis* grow in shady, deciduous forests of tropical Africa (Lundgren *et al.*, 2015), and the recently identified intermediates in *Homolepis* (Khoshravesh *et al.*, 2016) grow at the margins of South American rainforests. These disparate characterizations urge a careful, data based evaluation of the C_3_–C_4_ niche, its variation among evolutionary lineages, and its relation to that of C_3_ and C_4_ relatives.

In this study, we use available global occurrence data and local habitat descriptions to characterize the niche of C_3_–C_4_ lineages, along with their close C_3_ and C_4_ relatives. The ecological data are used to (i) quantitatively and objectively describe the abiotic habits of C_3_–C_4_ taxa and determine whether they inhabit uniform conditions, (ii) test whether phylogenetic effects partially explain the ecological sorting of C_3_–C_4_ lineages and whether their sorting explains the diversity in the ecology of C_4_ relatives, and (iii) test whether, when controlling for phylogenetic effects, the C_3_–C_4_ physiology affects the niche, potentially bringing the plants closer to the C_4_ niche. Our large-scale analyses, which consider all described C_3_–C_4_ lineages and their relatives, show that C_3_–C_4_ plants inhabit a large array of habitats, and that physiology closely interacts with evolutionary history to shape the niches of C_3_–C_4_, but also C_4_, taxa.

## Methods

### Ecological distribution of individual C_3_–C_4_ species

A list of 56 C_3_–C_4_ intermediate taxa was assembled from the literature, and included 11 eudicot and two monocot families (Table 1). Occurrence data for each taxon were downloaded from the Global Biodiversity Information Facility (GBIF, http://www.gbif.org) using the RGBIF package in R (Chamberlain *et al.*, 2016; data accessed 1 and 2 July 2016). Occurrence data for the Zambezian C_3_–C_4_ within *Alloteropsis semialata* were taken from Lundgren *et al.* (2015, 2016). All occurrence data were cleaned by removing any anomalous latitude or longitude points, points falling outside of a landmass, and any points close to GBIF headquarters in Copenhagen, Denmark, which may result from erroneous geolocation. To avoid repeated occurrences, latitude and longitude decimal degree values were rounded to two decimal places, and any duplicates at this resolution were removed. These filters are commonly applied to data extracted from GBIF (Zanne *et al.*, 2014).

**Table 1.** Details of C_3_–C_4_ taxa used in this study and their local habitats

Comparison	Species	*n*	Habitat	Reference^*a*^
Acanthaceae				
*Blepharis*	*B. diversispina*	42	Deciduous woodland, grasslands, soil sandy and gravelly, disturbed	Fisher *et al.*, 2015; Hyde *et al.*, 2016*a*,b; USDA/NRCS, 2016
	*B. gigantea*	6	Sandy to stony soils	
	*B. natalensis*	6	Rocky slopes	
	*B. noli-me-tangere*	2	Sandy soil, dry watercourses	
	*B. pruinosa*	19	Sandy to stony soils	
	*B. sinuata*	4	Bushland	
	*B. espinosa*	5	Deciduous woodland, disturbed, various habitats	
Amaranthaceae				
*Alternanthera*	*A. ficoidea*	268	Uplands	Rajendrudu *et al.*, 1986
	*A. tenella*	446		
*Salsola*	*S. divaricata*	32	Semi-arid rocky zones near coastal areas; salt tolerant	Voznesenskaya *et al.*, 2013
*Sedobassia*	*S. sedoides*	3	Ruderal, sandy, saline habitats	Eliáš and Dítě, 2014; Freitag and Kadereit, 2014
Asteraceae				
*Flaveria*	*F. pubescens*	8	Wetlands, alkaline and saline soils, fine textured soils	Powell, 1978; Edwards and Ku, 1987; USDA/NRCS, 2016
	*F. oppositifolia*	36		
	*F. angustifolia*	16	Pastures, fields, roadsides, disturbed	
	*F. anomala*	44		
	*F. chloraefolia*	16	Wetlands, saline and gypseous soils, disturbed	
	*F. floridana*	3	Wetlands, woodlands, sandy, saline, disturbed	
	*F. linearis*	77	Wetlands, woodlands, sandy, disturbed	
	*F. ramosissima*	6		
	*F. sonorensis*	3	Disturbed, semiarid soils	
*Parthenium*	*P. hysterophorus*	11	Disturbed, mainly dry or saline soils	Hedge and Patil, 1980; Moore *et al.*, 1987
Boraginaceae				
*Heliotropium*	*H. convolvulaceum*	164	Sand dune specialist	Frohlich, 1978; Vogan *et al.*, 2007
	*H. lagoense*	5		
	*H. greggii*	49	Open site, lay, gravel soils	
Brassicaceae				
*Diplotaxis*	*D. erucoides*	2328	Disturbed	Apel *et al.*, 1997; USDA/NRCS, 2016
	*D. muralis*	4828	Grazed grasslands, disturbed	
	*D. tenuifolia*	7206	Wetlands, wet woods, mountain slopes, sandy, disturbed	
*Moricandia*	*M. nitens*	285		Holaday and Chollet, 1984; USDA/NRCS, 2016
	*M. sinaica*	14		
	*M. spinosa*	1		
	*M. suffruticosa*	32		
	*M. arvensis*	821	Grainfields, orchards, disturbed	
Cleomaceae				
*Cleome*	*C. paradoxa*	7	Arid, rocky soils	Voznesenskaya *et al.*, 2007; Feodorova *et al.*, 2010
Euphorbiaceae				
*Euphorbia*	*E. acuta*	7	Dry limestone uplands, semi-arid scrublands, disturbed	Sage *et al.*, 2011
	*E. johnstonii*	1	Dry limestone uplands, semi-arid scrublands; calcareous soils, caliche outcrops	
	*E. lata*	52	Dry limestone uplands, semi-arid scrublands; calcareous soils, sandy plains	
Molluginaceae				
*Hypertelis*	*Hypertelis spergulacea*	16		Edwards and Ku, 1987; Christin *et al.*, 2011*b* ; USDA/NRCS, 2016
	*Paramollugo nudicaulis*	203	Ruderal habitats lacking competition	
*Mollugo*	*M. verticillata*	1686	Fields, gardens, disturbed, moist to dry soils; lacking competition	
Portulacaceae				
*Portulaca*	*P. cryptopetala*	35	Moist, warm habitats	Voznesenskaya *et al.*, 2010
Scrophulariaceae				
*Anticharis*	*A. ebracteata*	5	Quartz gravel	Khoshravesh *et al.*, 2012
	*A. juncea*	7	Farm, granite rocks	
Cyperaceae				
*Eleocharis*	*E. atropurpurea*	355	Wetlands, disturbed	Roalson *et al.*, 2010; USDA/NRCS, 2016
	*E. brainii*	6		
	*E. flavescens*	182	Wetlands	
	*E. nigrescens*	53	Wetlands, woodlands, sandy and peaty soils	
	*E. subfoliata*	6		
Poaceae				
*Alloteropsis*	Zambezian *A. semialata*	13	Shady, miombo woodlands	Lundgren *et al.*, 2015; 2016
*Homolepis*	*H. aturensis*	411	Rainforest	Khoshravesh *et al.*, 2016
*Neurachne*	*N. minor*	69	Arid soils, often shallow	Prendergast and Hattersley, 1985; Hattersley *et al.*, 1986
*Steinchisma*	*S. cuprea*	8		Edwards and Ku, 1987; USDA/NRCS, 2016
	*S. decipiens*	130		
	*S. hians*	285	Wetlands	
	*S. spathellosum*	57		
	*S. stenophyllum*	6	Wetlands	

^*a*^ References describe local habit. Those characterising C_3_–C_4_ intermediate status are italicized.

Environmental parameters that have been predicted to potentially explain the sorting of C_3_, C_3_–C_4_, and C_4_ photosynthetic types were selected (Christin and Osborne, 2014; Supplementary Table S1). Geographic distributions are characterized with latitudinal and altitudinal ranges, and broad climatic distributions are characterized via mean annual precipitation (MAP) and mean annual temperature (MAT) variables. The growing season temperature (i.e. temperature of the wettest quarter), minimum temperature (i.e. minimum temperature of the coldest month), number of annual frost days, minimum precipitation (i.e. precipitation of the driest month), number of annual wet days, percentage of maximum possible sunshine, rainfall seasonality, and fire return interval (FRI) were also used to characterize the environment. The rainfall seasonality data, which come from Lehmann *et al.* (2011), are based on an index that indicates how evenly dispersed rainfall is throughout a year, with zero indicating equal rain in all months and a value of 100 indicating that all annual rain fell within a single month (see Supplementary Table S1). The FRI data, which come from Archibald *et al.* (2013), are based on an index that indicates the growth time available to plants between fires, with greater FRI values indicating less frequent fire regimes and longer regrowth periods (Supplementary Table S1). Climate and soil fertility data were obtained by overlaying the occurrence coordinates onto high-resolution raster layers obtained from WorldClim (http://www.worldclim.org; Hijmans *et al.*, 2005), Climatic Research Unit (New *et al.*, 2002; http://www.ipcc-data.org), and the Harmonized World Soils Database (HWSD; FAO/IIASA/ISRIC/ISSCAS/JRC, 2012; http://webarchive.iiasa.ac.at; Supplementary Table S1).

Data from the dominant soil type of the topsoil layer were extracted from the HWSD raster layers. Specifically, four soil parameters were used to characterize soil fertility and are described below as per the HWSD classifications (FAO/IIASA/ISRIC/ISSCAS/JRC, 2012). First, the percentage of organic carbon (OC) in the topsoil is a particularly good indicator of soil health, with moderate to high OC present in fertile, well-structured soils. Soils with less than 0.2% or 0.6% OC are considered very poor and poor, respectively, and soils with greater than 2% OC are considered fertile. Total exchangeable bases (TEB) is the sum of exchangeable cations of sodium, calcium, magnesium, and potassium in the topsoil and, as such, soils with more TEB have better fertility. The cation exchange capacity (CEC) of the topsoil indicates the total nutrient fixing capacity of the soil, with low CEC soils, such as sandy soils with CEC less than 4 cmol kg^–1^, having little resilience and low nutrient stores, while soils with greater than 10 cmol kg^–1^ have high nutrient fixing capacity and are suitable for crops. The pH of the topsoil indicates the acidity and alkalinity of the soil, with pH values less than 4.5, as found in mangrove soils or acid sulfate soils, being extremely acid and poorly draining, pH values of 5.5–7.2 are considered neutral, and those above 8.5 are alkaline and consequently may inhibit the bio-availability of nutrients in the soils.

The variation among environmental variables at individual plant occurrence points was summarized using a principal component analysis (PCA), as implemented in the FactoMineR package in R (Lê *et al.*, 2008). A first PCA was conducted on climate variables, as described in Lundgren *et al.* (2015), and a second PCA was completed on the four soil fertility variables.

### Testing for phylogenetic effects on the ecological sorting of C_3_–C_4_ lineages

To determine whether the ecological sorting of C_3_–C_4_ taxa is partially determined by the phylogenetic lineage to which they belong, we tested for an effect of the abiotic environment of the closest C_3_ relatives on the sorting of C_3_–C_4_ lineages, and for an effect of the C_3_–C_4_ habitat on the sorting of the C_4_ relatives. For this purpose, we identified sets of C_3_–C_4_ species and their C_3_ and C_4_ sister groups. An angiosperm-wide phylogeny including all of the C_3_–C_4_ groups and their relatives was unavailable, and thus groups were defined based on phylogenetic trees published for the different clades (see Supplementary Table S2). This endeavor was complicated by taxa with unknown photosynthetic types. In addition, while some small groups have well resolved phylogenetic trees with clearly identified photosynthetic types (e.g. *Flaveria*; McKown *et al.*, 2005), many other systems have only been partially sampled or phenotyped. Nodes separating clearly identified C_3_ and C_3_–C_4_, or C_3_–C_4_ and C_4_ groups were selected, ignoring any groups with unknown photosynthetic types. For some C_3_–C_4_ lineages, either the C_3_ or the C_4_ sister group could not be identified. For example, *Portulaca cryptopetala* is nested in a group of C_4_ species and the related species are potentially CAM (Ocampo and Columbus, 2010; Arakaki *et al*., 2011), and several C_3_–C_4_ intermediates lack close C_4_ relatives (Supplementary Table S2). In cases where C_3_–C_4_ taxa were mixed with species of unknown type, the C_3_–C_4_ taxa were grouped and compared with a more distant clade with clearly established C_3_ taxa (e.g. *Eleocharis*; Roalson *et al.*, 2010; *Paramollugo*; Christin *et al.*, 2011*b*), and C_3_–C_4_ groups forming paraphyletic clades with respect to C_4_ species were merged (e.g. *Flaveria*; McKown *et al.*, 2005; Lyu *et al.*, 2015). However, C_3_–C_4_ belonging to the same family, but with distinct C_3_ and C_4_ relatives were considered separately (Supplementary Table S2). In other cases, where the phylogeny or photosynthetic categorization for a genus was incomplete, only taxa with clearly assigned photosynthetic types were considered and grouped based on the photosynthetic type independently of the phylogenetic relationships (e.g. *Heliotropium*; Supplementary Table S2). This approach decreases the number of contrasts, as closely related, yet independent C_3_–C_4_ lineages might have been merged. However, it ensures that no erroneous comparisons are included, for example when available plastid phylogenies do not perfectly match genome-wide relationships (e.g. Lyu *et al.*, 2015). Indeed, our analyses only compare photosynthetic types within groups that are monophyletic, even if these are incompletely sampled. In conclusion, while the incomplete phylogenetic knowledge probably decreases our analytical power, our approach is statistically conservative.

The abiotic environment of C_3_ and C_4_ relatives of C_3_–C_4_ lineages was assessed as described for C_3_–C_4_ taxa. For each species and each variable, the median was used to avoid extreme values, which could be misidentifications or erroneously reported occurrence points. To obtain one value per group, the average of the medians was calculated for each C_3_–C_4_ lineage, its C_3_ sister group, and its C_4_ sister group. A phylogenetic effect on the sorting of C_3_–C_4_ taxa was evaluated with correlation tests between the climatic environment of the C_3_ group and the environment inhabited by its closely related C_3_–C_4_ group. In the absence of phylogenetic effects, the values for C_3_–C_4_ taxa should be independent from those observed in the closely related C_3_ group. These analyses were repeated by testing for a correlation between the environment of the C_3_–C_4_ lineage and that of the closely related C_4_ group. Because many variables failed the Shapiro–Wilk test for normality, correlations were tested using the non-parametric Kendall rank correlation, which does not assume normality and is unbiased by small sample sizes. These tests were performed on the primary axis of the climate and soil PCAs, as well as on four climate variables (i.e. growing season temperature, minimum temperature, minimum precipitation, and rainfall seasonality) and two soil fertility variables (i.e. topsoil organic matter and TEB). These variables were selected to capture both temperature and precipitation patterns, which have classically been linked to photosynthetic types (reviewed in Christin and Osborne, 2014), and the two soil variables were selected to characterize the overall soil fertility. *P*-values of all tests were compared with a threshold corrected for eight comparisons (two PCA primary axes and six independent environmental variables; 0.00625).

### Testing for differences among photosynthetic types, while controlling for phylogeny

Phylogenetic effects and photosynthetic types can both potentially contribute to the ecological sorting of plants. We consequently tested for differences among photosynthetic types, while controlling for phylogenetic effects. A sister group approach was adopted to compare C_3_, C_3_–C_4_, and C_4_ photosynthetic types within each lineage (see Supplementary Table S2), an approach that removes phylogenetic effects in a similar manner to phylogenetic independent contrasts (Garland *et al.*, 1992). Indeed, a directional shift consistently associated with a given photosynthetic type within each group is strongly indicative of non-random processes (Vamosi and Vamosi, 2005; Edwards and Still, 2008; Edwards and Smith, 2010; Spriggs *et al.*, 2014). The age of the different groups varies (Christin *et al.*, 2011*a*, 2014), which means that the amount of divergence between the photosynthetic types is not necessarily constant among groups. However, our analyses are based on rank or sign tests and are therefore unaffected by variation in the magnitude of differences between photosynthetic types within each group. Consistent shifts between photosynthetic types were evaluated as the number of clades where the mean of the medians of the type of interest (either C_3_–C_4_ or C_4_) was larger than the mean of the medians of the comparison (C_3_ and C_3_–C_4_, respectively). The probability of observing such a shift with a random process (i.e. a probability of success of 0.5) was calculated based on a binomial distribution, in a two-tailed sign test. These tests were performed on the same eight variables used to assess the phylogenetic effects on C_3_–C_4_ sorting, and using the same corrections for multiple testing.

## Results

### Geographic distribution of C_3_–C_4_ intermediates

**Fig. 1.**
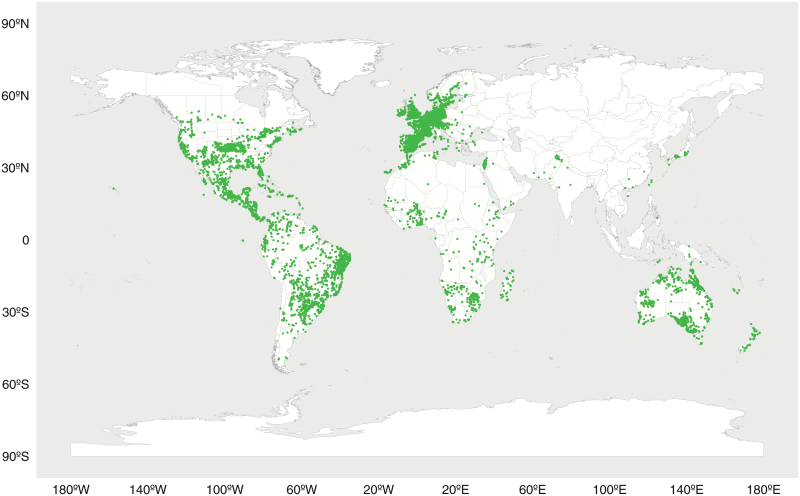
Global distribution of C_3_–C_4_ taxa. Each dot represents an occurrence point for a single C_3_–C_4_ intermediate plant.

As a whole, C_3_–C_4_ intermediates are broadly distributed across Australia, Asia, Europe, Africa, and the Americas (Fig. 1). While the sampling is clearly biased toward western Europe, Central America, and specific countries (e.g. Israel), the data clearly indicate that intermediates can occur in most tropical and temperate regions. The C_3_–C_4_ occurrences span a latitudinal belt between 50°S and 65°N (Fig. 1, Table 2, and Supplementary Dataset S1), with *Diplotaxis* intermediates reaching from northern Europe to the south of Australia, Africa, and South America (see Supplementary Fig. S1). *Eleocharis* and *Mollugo* C_3_–C_4_ plants are similarly widespread, spreading across the Americas, Europe, Africa, Asia, and Australia (Table 2, Supplementary Figs S1 and S2, and Supplementary Dataset S1). Other intermediate lineages, such as *Alloteropsis*, *Neurachne*, *Blepharis*, and *Sebodassia*, have smaller geographic ranges, according to the available occurrence data (Table 2, Supplementary Figs S1 and S2, and Supplementary Dataset S1). Many intermediates occur well below sea level, along the Dead Sea (e.g. *Diplotaxis erucoides*, *Moricandia sinaica*, and *Parthenium hysterophorus*), in The Netherlands (e.g. *Diplotaxis tenuifolia* and *Diplotaxis muralis*), or along the Gulf of Mexico (e.g. *Flaveria linearis*, *Eleocharis atropurpurea*; Table 2, Supplementary Figs S1 and S2, and Supplementary Dataset S1). C_3_–C_4_ intermediates also occur at high elevations, along the Andes mountains (e.g. *Steinchisma decipiens*, *Steinchisma hians*, *Mollugo verticillata*, *Diplotaxis muralis*), in Lesotho (e.g. *Diplotaxis muralis*, *Blepharis espinosa*), and in the highlands of Mexico (e.g. *Mollugo verticillata*, *Berkheya spinosissma*; Table 2; Supplementary Figs S1 and S2; Supplementary Dataset S1).

**Table 2.** Ranges of geography, climate, and soil characteristics of C_3_–C_4_ taxa within each lineage group

C_3_–C_4_ group	*n*	Latitude	Altitude (m)	MAT (°C)	MAP (mm)	OC (% weight)	TEB (cmol/kg)	CEC (cmol/kg)	pH (–log (H^+^))
Eudicots									
* Alternanthera*	714	35°S–51°N	0–2873	8–29	363–4523	0.1–16	0.2–76	1–76	3.3–8.4
* Anticharis*	12	29°S–22°S	289–1831	18–23	27–442	0.3–0.7	1.5–16	2–16	5.5–8.6
* Blepharis*	84	33°S–12°S	182–2555	10–23	100–1228	0.1–1.6	0–41	0–41	4.9–9.8
* Cleome*	7	11°N–16°N	23–777	25–29	38–503	0.3–0.7	6.8–17	6–17	6.5–8.1
* Diplotaxis* ^*a*^	14362	50°S–65°N	–409 to 3959	–2 to 26	33–2990	0.1–39.4	0.8–68.2	1–87	4.1–8.8
* Euphorbia*	60	25°N–38°N	59–1913	11–23	245–736	0.4–1.8	4.4–31.1	5–23	6.0–8.4
* Flaveria*	209	17°N–35°N	–1 to 3116	10–27	214–1581	0.3–14	1.7–83	4–83	4.5–8.4
* Heliotropium*	218	15°S–40°N	0–2543	9–26	63–2183	0.1–14	1.1–44	2–44	4.7–8.4
* Hypertelis*	16	29°S–28°S	68–1086	16–23	41–98	0.4–0.7	4.0–16	4–16	6.5–8.5
* Mollugo* ^*a*^	1889	38°S–53°N	–5 to 4209	0–30	1–4048	0.1–35.3	0.2–83	2–85	3.3–10.2
* Moricandia* ^*a*^	1153	35°S–60°N	–251 to 2701	6–25	10–1328	0.2–2.7	2.0–46.6	3–43	4.4–8.7
* Parthenium* ^*a*^	11	22°S–33°N	–228 to 904	18–23	325–1685	0.4–1.6	1.7–45.2	6–44	4.9–8.1
* Portulaca*	35	34°S–17°S	2–1948	15–26	308–1749	0.4–2.5	0.6–43.4	2–43	4.9–9.0
* Salsola*	32	28°N–40°N	5–1066	14–21	97–545	0.5–1.4	4.5–24.3	5–16	6.4–8.0
* Sedobassia*	3	43°N–48°N	64–97	10–12	527–540	1.1–1.7	38.0–40.9	23–43	6.9–7.8
Monocots									
* Eleocharis*	604	35°S–51°N	–1 to 3805	–1 to 29	163–4614	0.1–35.3	0.2–76	2–84	3.3–8.9
* Alloteropsis*	13	13°S–6°S	958–2264	18–24	812–1439	0.7–2.5	0.8–12	5–20	4.6–6.5
* Homolepis*	411	18°S–20°N	0–3548	8–28	671–7731	0.1–28	0.2–83	1–85	3.3–8.3
* Neurachne*	69	34°S–23°S	205–637	14–24	166–1128	0.3–2.1	2.1–18.1	2–15	4.5–8.3
* Steinchisma* ^*a*^	486	35°S–37°N	2–4524	3–27	229–3104	0.2–5.3	0.2–45.2	2–46	3.5–9

CEC, topsoil cation exchange capacity; MAP, mean annual precipitation; MAT, mean annual temperature; OC, topsoil organic matter content; TEB, topsoil total exchangeable bases.

^*a*^ C_3_–C_4_ lineages lacking close C_4_ relatives.

### Environmental distribution of C_3_–C_4_ intermediates

**Fig. 2.**
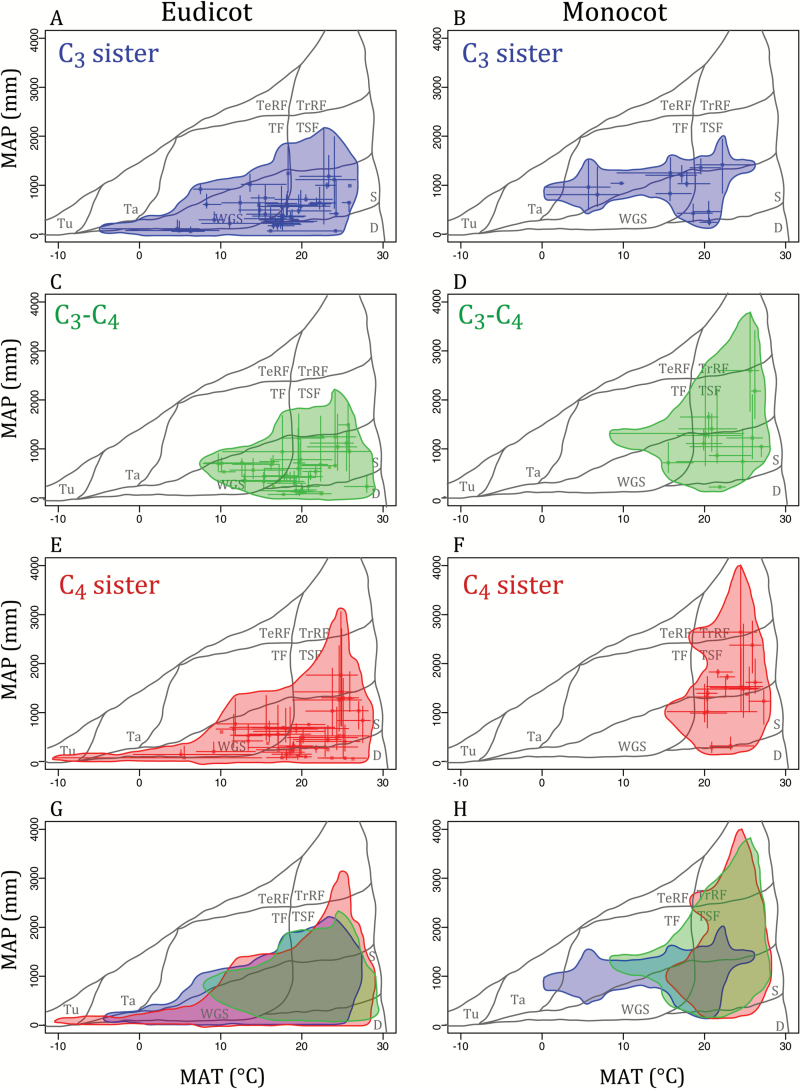
Comparative C_3_–C_4_ distributions across biomes. The median ± 10th and 90th quantiles for mean annual temperature (MAT) and precipitation (MAP) are plotted for eudicot (left) and monocot (right) C_3_ sister (blue; A, B), C_3_–C_4_ (green, C, D), and C_4_ sister (red, E, F) taxa. The bottom row overlaps the three distributions for eudicots (left, G) and monocots (right, H). All panels contain biome classifications (see Ricklefs, 2008) for tropical rainforest (TrRF), temperate rainforest (TeRF), temperate forest (TF), tropical seasonal forest (TSF), woodland/grassland/shrubland (WGS), savanna (S), desert (D), taiga (Ta), and tundra (Tu).

As a whole, C_3_–C_4_ taxa are broadly distributed across environments, inhabiting a variety of warm biomes, from tropical rainforests to deserts (Fig. 2C, D and Tables 1 and 2). In particular, C_3_–C_4_ eudicots are distributed within tropical seasonal forests, savannas, the woodland/grassland/shrubland habitats, temperate forests, and deserts (Fig. 2C, G). C_3_–C_4_ monocots are primarily distributed within tropical seasonal forests and savannas (Fig. 2D, H). Unlike C_3_–C_4_ eudicots, they are largely excluded from deserts and are present in tropical rainforests. They also have a smaller presence in the woodland/grassland/shrubland habitats than eudicot intermediates (Fig. 2D, H).

While the exact conditions in which the plants grow are not captured by average climatic variables, especially for annuals, annual precipitation may be virtually absent (e.g. *Mollugo verticillata* in the warm coastal deserts of Peru) or over 7700 mm (e.g. *Homolepis aturensis* in the tropical rainforests of Colombia) in habitats supporting C_3_–C_4_ intermediates (Table 2 and Supplementary Dataset S1). C_3_–C_4_ plants can inhabit regions with mean annual temperatures just below zero (e.g. *Diplotaxis muralis*, *Diplotaxis tenuifolia*, *Eleocharis flavescens*), but also as high as 30°C (e.g. *Paramollugo nudicaulis*; Table 2 and Supplementary Dataset S1). They exist in areas with winter temperatures down to –25°C (e.g. *Diplotaxis muralis* and *Mollugo verticillata* in Ontario and Saskatchewan, Canada) and 285 days of frost per year (e.g. *Mollugo verticillata* in the Rocky Mountains of Colorado and *Eleocharis flavescens* in the Andes of Chile) and growing season temperatures as low as –10°C (e.g. *Eleocharis flavescens* in Wyoming) but also above 32°C (e.g. *Paramollugo nudicaulis* in Pakistan, *Heliotropium convolvulacea* in California, *Eleocharis atropurpurea* in Western Australia, and *Cleome paradoxa* in Ethiopia; Supplementary Dataset S1). These broad climatic variables do not encapsulate the micro-environment of each species. Of the plants that inhabit the coldest climates, *Mollugo verticillata* and *Diplotaxis muralis* are annuals, and the perennial *Eleocharis flavescens* occurs in aquatic environments connected to warm thermal water (Simpson and Simpson, 2015). However, these regional climatic variables do highlight the broad-scale variation among C_3_–C_4_ taxa. The broad ecological distribution of C_3_–C_4_ taxa found in the global raster datasets is supported by species-specific habitat descriptions from the literature (Table 1). These descriptions report C_3_–C_4_ plants from deciduous woodlands, grasslands, wetlands, scrublands, and mountainous slopes, as well as from a variety of soil types (e.g. from fine-textured, to sandy, gravelly, and rocky soils; Table 1).

### Phylogenetic effects on the sorting of C_3_–C_4_ taxa and their C_4_ relatives

**Fig. 3.**
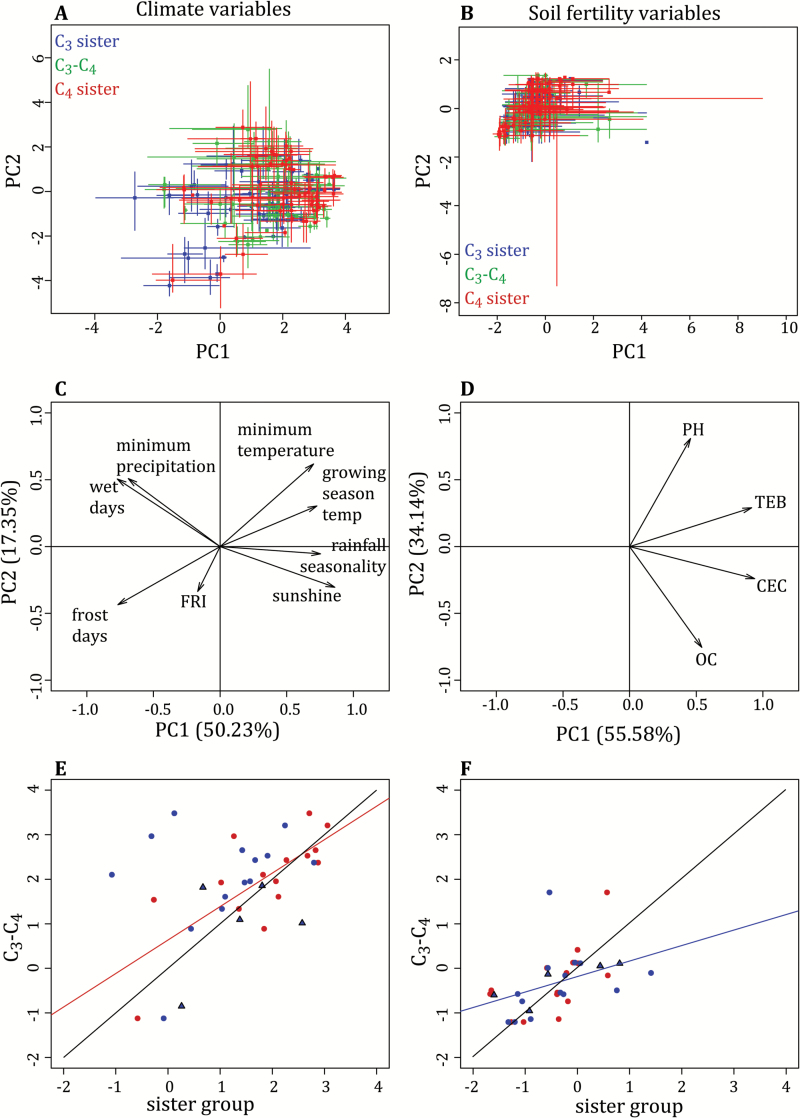
Distribution of photosynthetic types in ecological space. The median ± 10th and 90th quantiles for the first two principal component axes (PC1 and PC2) of the climate (A) and soil fertility (B) PCAs for C_3_ sister (blue), C_3_–C_4_ (green), and C_4_ sister (red) taxa. The associated variable factor maps for the climate and soil fertility PCAs are shown in (C, D). Shifts in the primary axis of the climatic (E) and soil fertility (F) PCAs, as comparisons between C_3_–C_4_ taxa and their closely related C_3_ (blue) and C_4_ (red) sister taxa within each phylogenetic group. Comparisons of C_3_–C_4_ taxa and their C_3_ relatives in groups that lack close C_4_ relatives are presented as blue triangles. Black lines indicate the 1:1 relationship. Linear relationships are shown for correlations significant after correction for multiple testing (*P*<0.00625), in the relevant color (see Table 3).

**Fig. 4.**
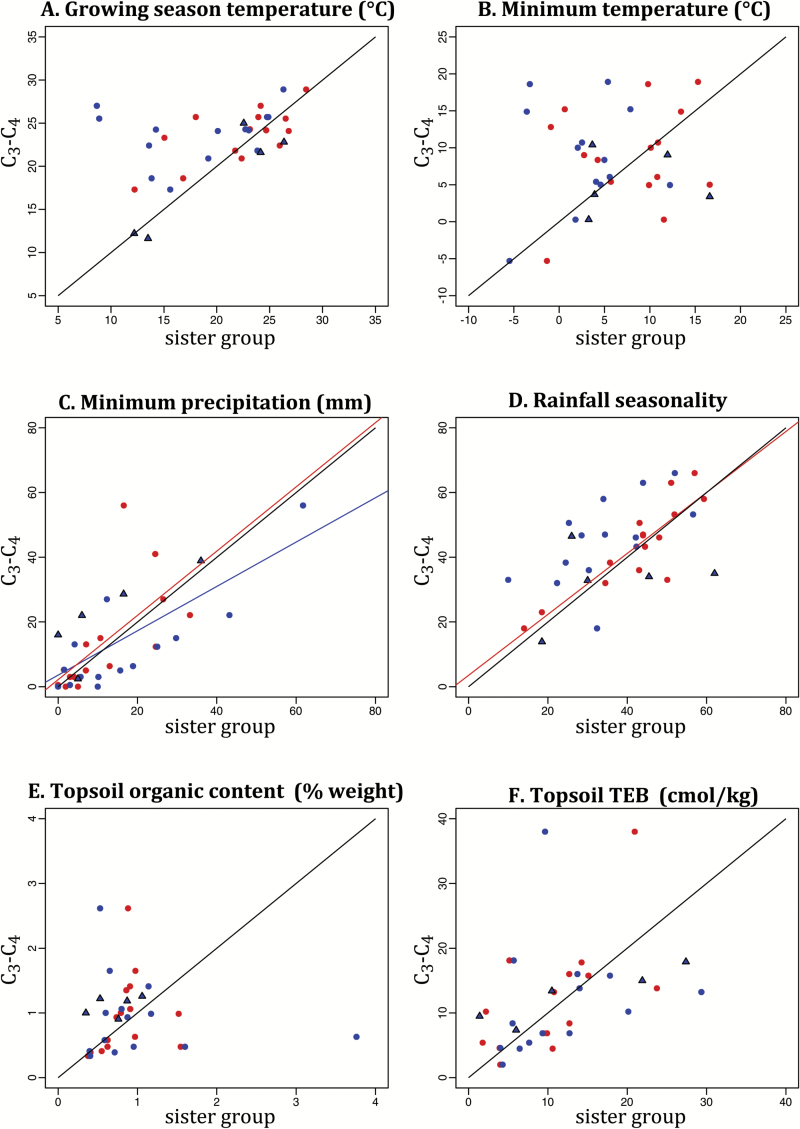
Ecological shifts between photosynthetic types. Shifts in growing season temperature, minimum temperature of the coldest month, minimum precipitation of the driest month, rainfall seasonality, topsoil organic matter content, and topsoil total exchangeable bases (as labelled) between C_3_–C_4_ taxa and their C_3_ (blue) and C_4_ (red) close relatives were evaluated. Each point represents an average for all species within each comparison group (see Methods). Comparisons of C_3_–C_4_ taxa and their C_3_ relatives in groups that lack close C_4_ relatives are presented as blue triangles. Black lines indicate the 1:1 relationship. Linear relationships are shown for correlations significant after correction for multiple testing (*P*<0.00625), in the relevant color (see Table 3).

The C_3_ relatives of C_3_–C_4_ lineages occur in a variety of temperature regimes from dry habitats to moderately wet ones, a pattern that is similar in monocot and eudicot systems (Fig. 2A, B). The medians of the C_3_–C_4_ lineages are widely distributed along the primary PCA axis for climatic variables, which explains 50.23% of the variation, and these are not correlated to those of their close C_3_ relatives (Fig. 3A, C, E and Table 3). However, the soil fertility conditions experienced by C_3_–C_4_ plants, extracted from the primary PCA axis for soil variables, which explains 55.58% of the variation, are correlated to those of their C_3_ relatives, which might be driven by topsoil TEB (Fig. 3B, D, F and Tables 3 and 4). Similarly, variation in minimum precipitation experienced by C_3_–C_4_ lineages is correlated to that of closely related C_3_ lineages (Fig. 4C and Table 3), indicating a strong phylogenetic effect.

**Table 3.** Kendall correlation tests for environmental medians among photosynthetic types across angiosperms

	C_3_–C_4_*vs*. C_3_		C_4_*vs*. C_3_–C_4_	
Variable	*P*-value	tau	*P*-value	tau
Climate PCA axis 1	0.27	0.19	0.0059*	0.52
Soils PCA axis 1	0.0032*	0.50	0.02	0.46
Growth season temperature	0.14	0.25	0.03	0.42
Minimum temperature	0.78	–0.05	0.85	0.05
Minimum precipitation	0.0041*	0.48	0.0025*	0.59
Rainfall seasonality	0.07	0.31	0.0011*	0.63
Topsoil organic content	0.92	0.02	0.02	0.47
Total exchangeable bases	0.04	0.34	0.03	0.42

* Tests that were considered significant, using a threshold of 0.00625, which corresponds to a 0.05 threshold corrected for eight tests.

The close C_4_ relatives of C_3_–C_4_ plants exist along a broad range of temperatures in eudicots, but are restricted to warmer areas in monocot species, resulting in less overlap between photosynthetic types in the latter than in the former (Fig. 2E–H). The variation among C_4_ lineages on the first axis of the climate variable PCA is correlated with that of their C_3_–C_4_ relatives (Fig. 3A, E and Table 3), indicating an overall phylogenetic effect on the sorting of C_4_ lineages. The soil fertility conditions experienced by C_4_ plants, assessed with the PCA on soil variables, is weakly correlated to that of their C_3_–C_4_ relatives (Fig. 3B, F and Table 3). Among the individual variables, the minimum precipitation and rainfall seasonality experienced by C_4_ lineages are correlated to that of their C_3_–C_4_ relatives (Fig. 4C, D and Table 3). Moreover, the growth season temperature and topsoil properties of C_4_ lineages are also weakly correlated to those of their close C_3_–C_4_ relatives; however, these do not remain significant after correcting for multiple tests (Fig. 4A, E, F and Table 3). Thus, the precipitation, and possibly the temperature and soil fertility, preferences of C_4_ lineages depend, to varying degrees, on phylogenetic effects.

### Effects of photosynthetic types after correcting for phylogenetic signals

The five C_3_–C_4_ lineages without close C_4_ relatives do not behave in the same manner as the lineages that did evolve C_4_ photosynthesis. With the exception of *Eleocharis*, which contains aquatic plants that grow in warm waters within cold climates, four of these five lineages are those that occupy the coldest environments experienced by intermediate plants (Table 2) and are primarily in habitats with higher minimum precipitation than their C_3_ relatives (Fig. 4C). All five of these C_3_–C_4_ lineages inhabit areas with more organic soils than their close C_3_ relatives (Fig. 4E). These lineages without C_4_ relatives are also among the most widely distributed of all intermediates groups (i.e. *Diplotaxis*, *Mollugo verticillata*; Supplementary Figs S1 and S2), which likely reflects an ability to tolerate diverse ecological conditions.

Considering the C_3_–C_4_ lineages with close C_4_ relatives, their distributions are significantly shifted toward positive values of the primary axis of the climate variable PCA, which corresponds to drier and warmer environments, compared with their paired C_3_ relatives (Fig. 3A, C, E and Table 4). This shift is reflected within the individual variables, with C_3_–C_4_ lineages occupying regions with warmer growing season temperatures, higher minimum winter temperatures, and more seasonal rainfall patterns than their C_3_ relatives (Table 4). Therefore C_3_–C_4_ intermediates tend to inhabit relatively warm regions, regardless of the habitat in which their C_3_ relatives occur, while their preference for habitat aridity does depend on the minimum precipitation experienced by their C_3_ relatives (Fig. 4A–C and Table 4).

**Table 4.** Tests for environmental shifts among photosynthetic types across angiosperms

	C_3_–C_4_*vs*. C_3_ (all lineages)		C_3_–C_4_*vs*. C_3_ (only lineages with close C_4_ relatives)		C_4_*vs*. C_3_–C_4_	
Variable	Observed^*a*^	*P*-value	Observed^*a*^	*P*-value	Observed^*a*^	*P*-value
Climate PCA axis 1	14/19	0.019	12/14	0.0018*	8/15	0.61
Soil fertility PCA axis 1	10/19	0.65	8/14	0.42	6/15	0.61
Growth season temperature	14/19	0.019	13/14	0.00012*	5/15	0.30
Minimum temperature	13/19	0.064	12/14	0.0018*	8/15	0.61
Minimum precipitation	7/19	0.36	3/14	0.057	7/15	1
Rainfall seasonality	14/19	0.019	12/14	0.0018*	6/15	0.61
Topsoil organic content	11/19	0.36	6/14	0.79	5/15	0.30
Total exchangeable bases	8/19	0.65	5/14	0.42	6/15	0.61

^*a*^ The number of points higher in the focal group is indicated.

* Tests that were considered significant, using a threshold of 0.00625, which corresponds to a 0.05 threshold corrected for eight tests.

None of the studied environmental parameters, including both of the composite PCA variables and the six individual environmental variables, show a significant shift between close C_3_–C_4_ and C_4_ relatives (Table 4). Therefore, with the data available here, the C_4_ physiology is not linked to consistent ecological shifts when controlling for phylogenetic effects.

## Discussion

### A uniform C_3_–C_4_ niche does not exist

C_3_–C_4_ taxa are remarkably widespread across geographical and environmental space, maintaining the ability to exist in both typical C_3_ and C_4_ niches (Figs 1–3 and Supplementary Figs S1 and S2). It should be noted that the GBIF occurrence data, if anything, represent a subset of the total geographic range for each species and the realized geographical and environmental ranges of these taxa may be larger than presented here, especially for groups distributed in poorly sampled areas, such as Africa and southeast Asia. However, since related taxa tend to occur in similar regions, a sampling bias would likely affect the different photosynthetic types within a lineage to a similar degree, and the dataset is therefore still representative of the relative distribution of each type. Furthermore, several of the C_3_–C_4_ groups likely include more intermediate species than we present here, as we considered only those taxa for which the photosynthetic type has been assessed with confidence. For instance, the photosynthetic type of only one species within the genus *Homolepis* has been determined (Khoshravesh *et al.*, 2016), while the remaining five congeners have not yet been characterized. The same is true of *Eleocharis*, where several species have been characterized as only possible intermediates (Roalson *et al.*, 2010) and, as such, were not included in the study. Finally, it is unknown whether the various occurrences for each taxon are using the same photosynthetic type, or whether these vary intraspecifically across space or environments, as has been observed in the grass *Alloteropsis semialata* (Lundgren *et al.*, 2015, 2016), and suggested for other taxa (e.g. Khoshravesh *et al.*, 2012). When this variation had been reported but not clarified, the taxon was ignored, but in most cases, only a limited number of plants have been screened per species. With these caveats in mind, it is clear that the physiology of C_3_–C_4_ plants does not strongly restrict the migration of species geographically or into new environments.

### Evolutionary history influences the realized ecology

While differences between sister groups can result from shifts in either group, they do allow for comparisons among character states independent of phylogeny. Interestingly, these analyses clearly show that the precipitation niches of C_3_–C_4_ taxa are statistically correlated to those of their close C_3_ relatives, specifically with respect to minimum precipitation. This suggests that C_3_–C_4_ plants can occur in arid habitats if their C_3_ relatives are already adapted to do so, and not specifically as a result of the C_3_–C_4_ physiology. Similarly, statistical evidence indicates that soil preferences of C_3_–C_4_ are correlated to those of their close C_3_ relatives. C_3_–C_4_ physiology is only part of the attributes that a plant can use to tolerate environmental conditions, which tend to be similar among relatives (Christin and Osborne, 2014). These attributes, which can include life-history traits, growth strategies, and other non-photosynthetic characters, lead to a certain niche conservatism. Moreover, related taxa tend to occur within the same regions as a function of their biogeography, which increases the likelihood of being found in similar environments. Both precipitation variables are similarly correlated between C_3_–C_4_ and C_4_ relatives, likely explaining previously reported differences among C_4_ lineages in aridity preferences (Teeri and Stowe, 1976; Stowe and Teeri, 1978; Taub, 2000; Christin and Osborne, 2014). The influence of evolutionary history on the realized C_4_ niche could go beyond precipitation preference, as our data suggest that temperature and soil fertility between C_3_–C_4_ and closely related C_4_ groups are also associated, although this was not significant with our small species sampling.

### C_3_–C_4_ species shift closer to the C_4_ niche

In some cases, C_3_–C_4_ lineages emerged from groups that already inhabited warm climates, as reported in C_4_ grasses (Edwards and Smith, 2010), while in others cases, C_3_ relatives exist in cold areas (Fig. 3A, C). Independent of C_3_ ecology, the C_3_–C_4_ lineages occupy warm habitats, which might reflect the increased temperature tolerance conferred by the C_3_–C_4_ physiology (Schuster and Monson, 1990). Despite some C_3_–C_4_ taxa persisting in cold regions, the convergence of physiological intermediates in warmer areas, whether that be in wet forests or dry deserts, may have increased the likelihood of further transitions to a C_4_ state that occupies a similar temperature niche. Therefore, in terms of temperature, the C_3_–C_4_ state brings lineages into warmer habitats that should promote photorespiration and, thus, may encourage selection for C_4_ physiology, thereby representing a true bridge between the ancestral C_3_ state and C_4_ origins. As more detailed phylogenies and updated lists of C_3_–C_4_ species become available, further comparative work might be able to distinguish whether this happens via an increase in C_3_–C_4_ migrations toward warmer habitats or a decrease in their migrations outside of such habitats, since both scenarios would result in a concentration of C_3_–C_4_ lineages in warmer habitats than their C_3_ relatives.

While precipitation preferences vary tremendously across C_3_–C_4_ lineages as a function of evolutionary history, these intermediate lineages shifted toward habitats with more rainfall seasonality than their close C_3_ relatives, yet no consistent shift was observed between C_3_–C_4_ plants and their C_4_ relatives (Table 4). Phylogenetic models in grasses have previously reported that C_4_ origins were accompanied by consistent shifts into drier habitats (Edwards and Smith, 2010), a trend that we suggest is initiated in C_3_–C_4_ taxa. Direct measurements and modelling efforts have failed to identify increases in water-use efficiency in intermediates of *Flaveria*, which suggests that the C_3_–C_4_ advantage is mainly linked to carbon gain, not water saving (Monson, 1989; Vogan and Sage, 2011; Way *et al.*, 2014). However, the xylem architecture was altered during the transition from C_3_ to C_3_–C_4_ species in *Flaveria*, providing protection against cavitation and hence increased drought tolerance (Kocacinar *et al.*, 2008). Such alterations of leaf hydraulics, if consistently associated with the C_3_–C_4_ type, might explain their observed propensity to migrate to habitats with higher rainfall seasonality, habitats that would promote episodes of water limitations, potentially increasing the pressure for further evolutionary transitions to C_4_ photosynthesis (Osborne and Sack, 2012), especially in warm habitats where C_3_–C_4_ plants tend to occur.

### The fate of C_3_–C_4_ lineages lacking C_4_ relatives

Since all of the taxa included in this study still naturally occur in the wild, they have all persisted in a C_3_–C_4_ state since their early emergence from C_3_ ancestors, which is estimated to be as recent as 2 and as old as 20 Ma, depending on the group (Christin *et al.*, 2011*a*). However, most of the known C_3_–C_4_ lineages are related to some C_4_ groups, which prove that their ancestors had the ability, at least at some point, to produce C_4_ descendants. Clear exceptions include the closely related groups *Diplotaxis* and *Moricandia*, which belong to a large family completely lacking C_4_ taxa (Brassicaceae). While three other C_3_–C_4_ groups (*Steinchisma*, *Mollugo verticillata*, *Parthenium*) belong to families with C_4_ origins, which are included here for other C_3_–C_4_ groups (Poaceae, Molluginaceae, Asteraceae), they are sufficiently distant from any C_4_ group in their phylogenies that one cannot be sure whether their ancestors were able at any point to produce C_4_ descendants (Christin *et al.*, 2011*b*; Grass Phylogeny Working Group II, 2012). It is therefore reasonable to ask whether some attributes of these groups decreased the likelihood of C_4_ evolution. While genomics, anatomy, and physiology might play a role (Christin *et al.*, 2013; Bräutigam and Gowik, 2016), the ecology might also affect these evolutionary trajectories. For instance, C_3_–C_4_*Moricandia* occurs mainly in colder climates, which might decrease pressure for C_4_ evolution. Three of the other four C_3_–C_4_ groups lacking close C_4_ relatives are among the most widespread geographically (see Supplementary Figs S1 and S2), and these groups tend to occur in habitats with relatively high minimum precipitation and fertile soil. While none of these factors should prevent C_4_ evolution in itself, it is possible that the realization of the C_3_–C_4_ phenotype in these groups was successful enough to limit selective pressures for further transitions in photosynthesis.

## Conclusions

In this study, we present the first systematic description of the geographical and ecological distribution of C_3_–C_4_ intermediates. Our investigations reveal that C_3_–C_4_ taxa are found in a very large range of conditions and habitats, from dry deserts to tropical rainforests and cold wetlands. This variation is partially explained by evolutionary history, with C_3_–C_4_ lineages tending to grow in habitats with similar precipitation to their C_3_ relatives, a conservatism that is further reported onto C_4_ lineages. However, C_3_–C_4_ taxa inhabit warm climates, independent of the ancestral condition, and shift toward more seasonal rainfall habitats. Our findings indicate that the C_3_–C_4_ condition moves lineages into environments that promote photorespiration and, as such, may facilitate the evolution of a full C_4_ pathway. There is, in our dataset, no clear difference between C_3_–C_4_ and C_4_ in any of the environmental preferences. However, different C_4_ groups might shift in various directions or extend their niche in ways that are not universal across flowering plants as, for example, it has been suggested that C_4_ evolution was linked to different pressures in grasses and chenopods (Osborne and Freckleton, 2009; Kadereit *et al.*, 2012). While group-specific detailed analyses might reveal peculiarities of each lineage, our angiosperm-wide joint analysis of C_3_, C_3_–C_4_, and C_4_ groups helps to disentangle the ecological changes that happened during consecutive phases of C_4_ evolution. Indeed, shifts toward drier and warmer habitats occurred in C_3_–C_4_ lineages, but others, such as geographic expansions, might be specific to the C_4_ state. When detailed phenotype information becomes available for a larger number of taxa, similar analyses might identify the changes linked to each individual C_4_ component, bringing together anatomy, biochemistry, physiology, and evolutionary ecology.

## Supplementary data

Supplementary data are available at *JXB* online.

Dataset S1. Occurrence and environmental data for C_3_–C_4_ taxa and their close C_3_ and C_4_ relatives used in this study.

Fig. S1. Distribution of C_3_ sister (blue), C_3_–C_4_ (green), and C_4_ sister (red) taxa in eudicot comparison groups.

Fig. S2. Distribution of C_3_ sister (blue), C_3_–C_4_ (green), and C_4_ sister (red) taxa in monocot comparison groups.

Table S1. Details on the environmental data used in this study.

Table S2. Details of C_3_–C_4_ species used in this study and the C_3_ and C_4_ sister taxa within each comparison group.

## Supplementary Material

Supplementary_Figure_S1

Supplementary_Figure_S2

Supplementary_Table_S1

Supplementary_Table_S2

Supplementary_Dataset_S1

## Abbreviations:

CEC: cation exchange capacity
FRI: fire return interval
MAP: mean annual precipitation
MAT: mean annual temperature
OC: organic carbon
TEB: total exchangeable bases
PCA: principal component analysis.
