# Supplementary figures and images for: Despite phylogenetic effects, C3–C4 lineages bridge the ecological gap to C4 photosynthesis
Source: J Exp Bot. 2016 Dec 26;68(2):241–54. doi: 10.1093/jxb/erw451 (PMC5853900; doi:10.1093/jxb/erw451)

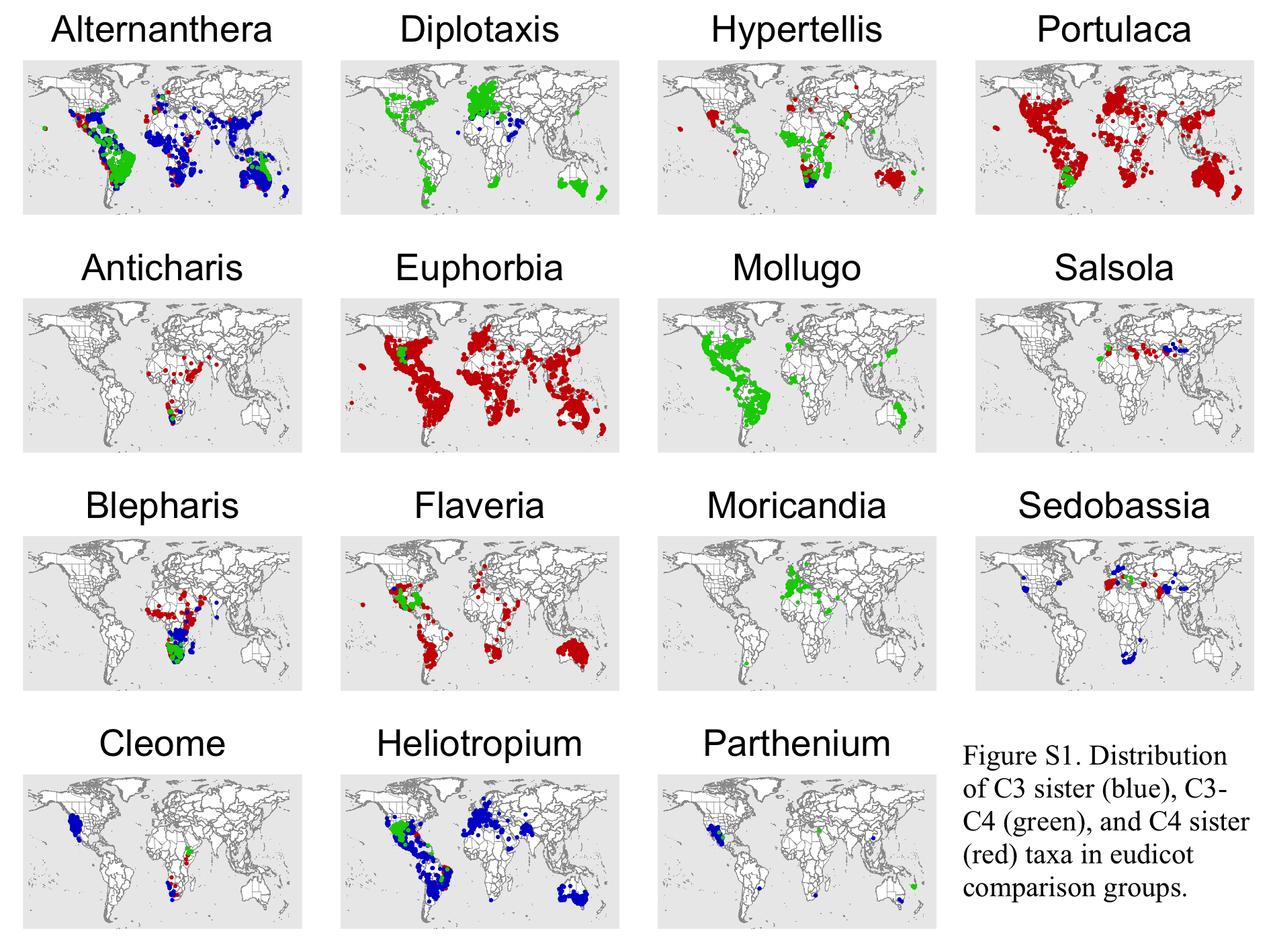

Supplement: Supplementary_Figure_S1 [file erw451_suppl_supplementary_figure_s1.png]

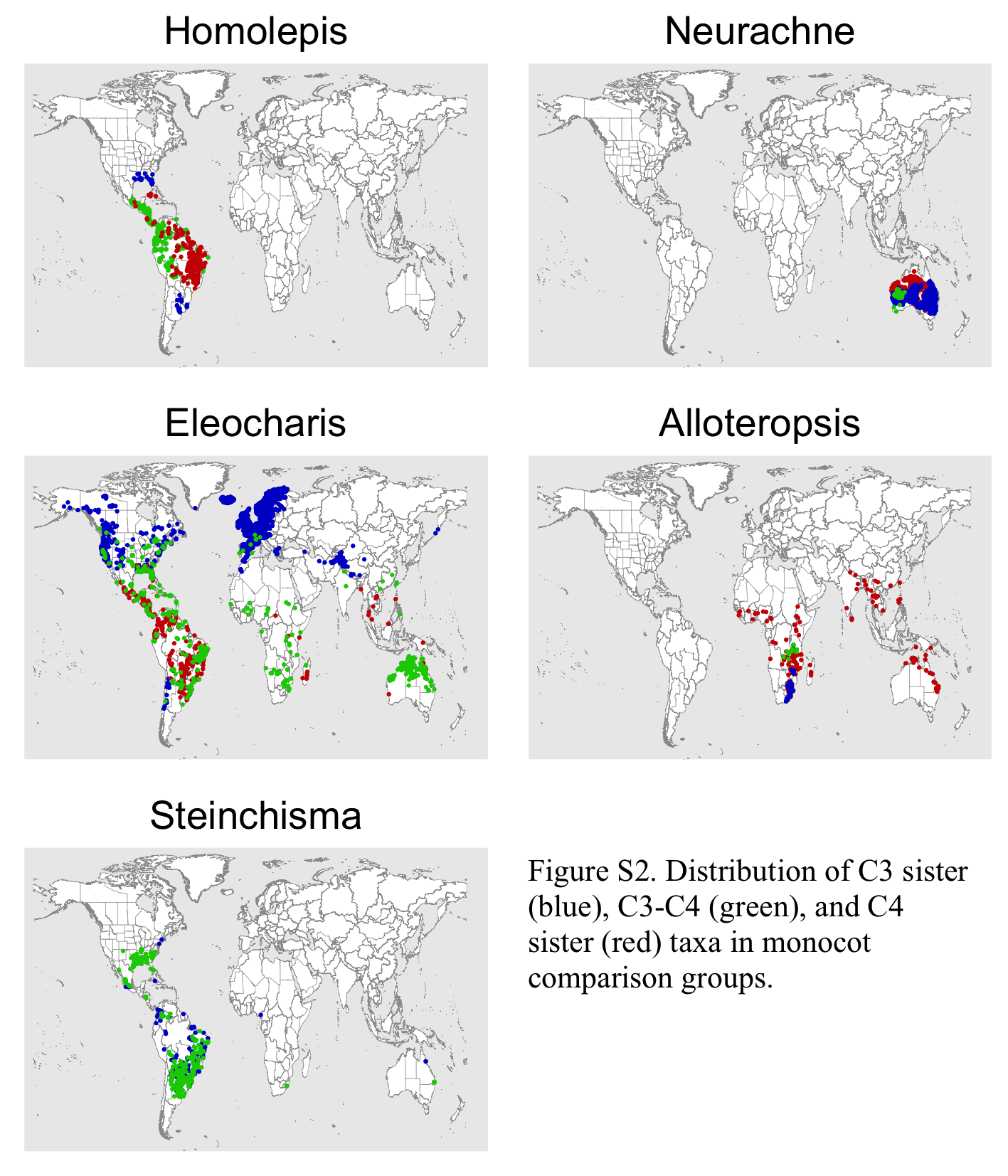

Supplement: Supplementary_Figure_S2 [file erw451_suppl_supplementary_figure_s2.png]
